# Demyelinating Neurological Adverse Events following the Use of Anti-TNF-*α* Agents: A Double-Edged Sword

**DOI:** 10.1155/2022/3784938

**Published:** 2022-03-07

**Authors:** Miral H. Gharib, Mohamed Awni AlKahlout, Beatriz Garcia Canibano, Dirk Theophiel Deleu, Hani Malallah AlEssa, Samar AlEmadi

**Affiliations:** ^1^Rheumatology, Department of Medicine, Hamad Medical Corporation, Doha, Qatar; ^2^Neurology, Department of Medicine, Hamad General Hospital, Doha, Qatar

## Abstract

**Background:**

Tumor necrosis factor antagonists (anti-TNF-*α*) are an established therapeutic option for several autoimmune and inflammatory bowel diseases. Despite their clinical effectiveness, neurological adverse events have been reported, and literature data suggest a potential role of anti-TNF-*α* in the induction of demyelination. *Case Presentation*. In this series, we present three cases of demyelination after the use of anti-TNF-*α* agents. The first case involved a 21-year-old man with HLA-B27 negative peripheral spondylarthritis who had been taking adalimumab for 2 years. He developed headache, urinary incontinence, and bilateral lower extremity numbness that progressed to the middle of the trunk for 2 days. Magnetic resonance imaging (MRI) showed multiple hyperintense enhancement lesions in the left paramedian anterior pons consistent with multiple sclerosis (MS). The second case included a 17-year-old woman who was on 2 years of adalimumab treatment for juvenile idiopathic arthritis and chronic anterior uveitis and developed new-onset dizziness and tremors. The clinical examination showed signs of cerebellar dysfunction. MRI findings were consistent with multiple sclerosis. The third case was a 34-year-old male who was on 5 years of infliximab treatment for ankylosing spondylitis when he developed left hand numbness and weakness. Cerebrospinal fluid (CSF) analysis and MRI findings were consistent with demyelination. Discontinuation of tumor necrosis factor antagonists (anti-TNF-*α*) resulted in resolution of the symptoms with no recurrence in the first case, but there was evidence of recurrence in the other 2 cases, where one was managed with rituximab and the second one improved with pulse steroid therapy.

**Conclusion:**

Despite the small number of patients, our series adds to the growing body of evidence supporting a causal link between anti-TNF-*α* agents and demyelination. Thus, we can conclude that on suspicion of any neurological side effects, early discontinuation of the TNF-*α* blockers and requesting urgent MRI scan to confirm the diagnosis is of utmost importance.

## 1. Introduction

Tumor necrosis factor-*α* (TNF-*α*) is a pleiotropic cytokine that plays a key role in host defense mechanisms and exhibits multifunctional proinflammatory properties [1]. It was found that the pathogenesis of many diseases, including rheumatoid arthritis, Crohn's disease, atherosclerosis, psoriasis, sepsis, diabetes, and obesity, has been associated with aberrant TNF-*α* development and TNF receptor signaling. Therefore, TNF-*α* and its receptor were proposed as a therapeutic target in the treatment of many diseases [2]. With great success in terms of efficacy and safety, anti-TNF-*α* agents have revolutionized the treatment of rheumatoid arthritis, ankylosing spondylitis, psoriasis, psoriatic arthritis, juvenile polyarticular rheumatoid arthritis, and inflammatory bowel disease. These agents act by abrogating the soluble TNF-*α*, thus preventing its binding on TNFR1/TNFR2 receptors [3, 4]. Currently, many anti-TNF-*α* agents have been licensed for treating certain inflammatory diseases including rheumatoid arthritis and inflammatory bowel disease. However, these agents are not without side effects. Aside from the usual presumed infectious difficulties, uncommon cases of neuroinflammatory demyelinating disorders, such as multiple sclerosis (MS), myelitis, and optic neuritis, have been documented with the use of anti-TNF-*α*, with a very low absolute risk below one in 1000 patients/year as recently shown in large Denmark and Sweden Cohort [5].

In our rheumatology division, around 500 patients are being treated with anti-TNF-*α* agents such as infliximab, adalimumab, and etanercept, which have been used widely specially in the treatment of rheumatoid arthritis, ankylosing spondylitis, and inflammatory bowel disease. However, their increasing use has revealed a variety of adverse events. We reported three cases of demyelination following the use of anti-TNF-*α*. The cases were diagnosed with MS based on McDonald's criteria (2017) for the diagnosis of multiple sclerosis [6]. These cases aimed at increasing the physician's awareness of the demyelinating adverse effect associated with anti-TNF-*α* therapy.

## 2. Case Presentation

**Figure 1:**
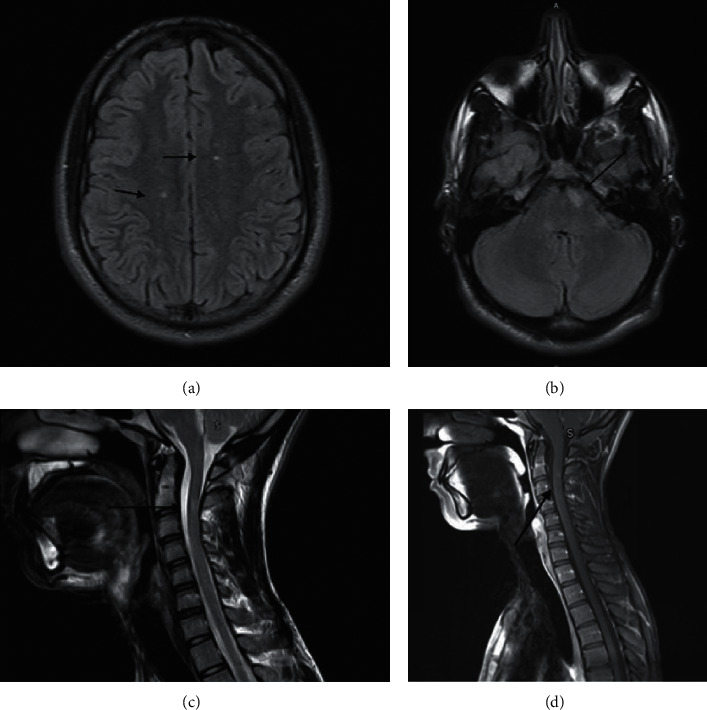
(a) There are few small foci of T2 and FLAIR hyperintensity noted in the periventricular white matter (bilaterally), perpendicular to the ventricular margin. (b) There is a small area of T2 and FLAIR hyperintensity noted in the left paramedian anterior pons. ((c), (d)) There is a small area of T2 hyperintensity with corresponding postcontrast enhancement noted in the cervical spinal cord at the C2-C3 level.

Case 1: a 21-year-old Qatari gentleman was on adalimumab for HLA-B27-negative peripheral spondyloarthritis. After being kept on adalimumab for 2 years, he developed severe headache, urinary incontinence, and bilateral lower limb numbness, which progressed towards the middle of the trunk over several days. The neurological examination uncovered dysesthesias below the dermatome T4 associated with brisk tendon reflexes, while the remainder of the examination was normal. Lumbar puncture was not done, and the diagnosis of multiple sclerosis (MS) was established by the presence of multiple hyperintense lesions on brain MRI in the left paramedian anterior pons, left posterolateral medulla, and bilateral periventricular white matter, some of them involving the optic radiations. Similar lesions were identified in the right lateral aspect of corpus callosum splenium and in the cervical spinal cord at the level of C2-C3 level and a small area of T2 hyperintensity in the thoracic spinal cord at the T5 level as shown in Figures 1(a)–1(d). There was no family history of MS.

Complete resolution of neurological symptoms due to discontinuation of adalimumab was observed within one month. So, no pulse steroid was given, and MRI was not repeated. However, he developed abdominal pain with loose bloody motions, in which ileocolonoscopy and biopsy confirmed the diagnosis of Crohn's disease. Remarkably, we found that he had a significant family ancestry of inflammatory bowel disease. His inflammatory bowel disease and peripheral arthritis were well-controlled on ustekinumab (IL12/23 inhibitor), and a 4 years follow-up showed no relapses.

**Figure 2:**
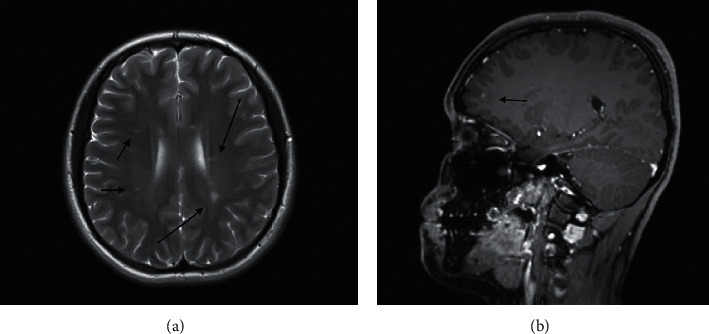
(a) There are multiple periventricular, subcortical, and juxtacortical white matter foci of T2 and FLAIR hypersignal in both cerebral hemispheres, brain stem, bilateral middle cerebellar peduncles, corpus callosum, and left anterior thalamus. (b) There is focal enhancement noted in the left peritrigonal white matter lesion.

Case 2: a 19-year-old Qatari woman, who was on adalimumab for juvenile idiopathic arthritis and chronic anterior uveitis for 2 years, developed new-onset dizziness and hand tremors. Neurological examination showed impaired cerebellar function as evidenced by intention tremors, ataxia, dysmetria, and dysdiadochokinesia with normal tone, power, and sensation; reflexes were less brisk. She had no history of multiple sclerosis. MRI revealed multiple hyperintense white matter lesions especially in bilateral cerebellar peduncles, periventricular, subcortical and juxtacortical, brain stem, corpus callosum, and left anterior thalamus, and also, multiple patchy T2 hyperintensities are noted in the cervical and midthoracic spinal cord with foci of enhancement noted in the midthoracic spinal cord, in keeping with active lesions as shown in Figures 2(a) and 2(b). Her CSF analysis revealed the presence of CSF unique oligoclonal bands.

Discontinuation of adalimumab resulted in near-complete improvement in her symptoms; she was offered pulse steroid, but she declined and was shifted to methotrexate with good control of her arthritis and uveitis. Repeated MRI after one year showed regression of the previous lesions, but it showed newly detected lesions in different areas. Subsequently, patient was switched to rituximab to control her arthritis and demyelinating disease. A year later, another MRI was done, and it showed stable appearance of the previous demyelinating lesions with no signs of disease activity.

**Figure 3:**
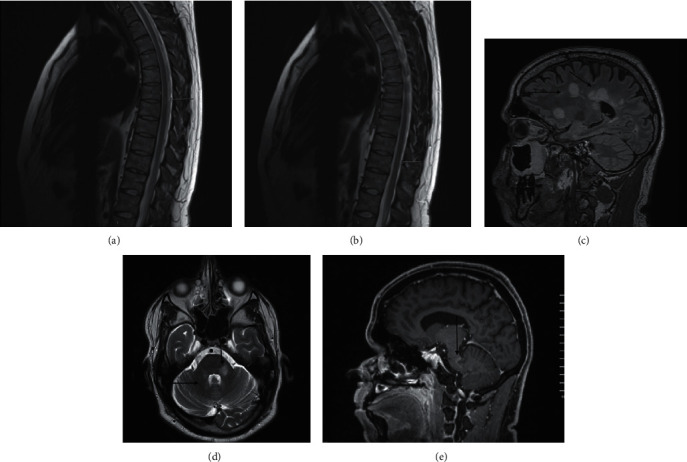
((a), (b)) There are at least 2 definite high signal intensity plaques on the sagittal T2W1 seen at D6-7, D9-10 levels (arrow). ((c), (d)) Numerous bilateral variable-sized, different-shaped plaques of high signal intensity on T2W1/FLAIR seen mainly in the deep white matter at periventricular location and centrum semiovale, perpendicular callososeptal lesions, cerebellar peduncles, and cerebellar white matter. (e) Incomplete ring enhancement lesions seen in the left deep periventricular frontal lobe and left cerebellar peduncle.

Case 3: a 34-year-old man was diagnosed with ankylosing spondylitis 7 years ago in his home country. He was initially treated with sulfasalazine and nonsteroidal anti-inflammatory drugs. After two years, he was started on infliximab to control his disease activity. Five years after infliximab initiation, he developed unexplained left hand numbness and weakness. A diagnosis of multiple sclerosis was made based on MRI findings and CSF analysis which were done at his home country. Infliximab was discontinued, and he received IV pulse steroid for five days with complete recovery; but two years later, he developed another attack with diplopia, left leg numbness, and weakness and again was treated with IV pulse steroids with good recovery. 4 years later, he presented to our hospital with a third attack of left facial numbness; MRI done at our center to confirm the diagnosis showed numerous bilateral variable-sized, different-shaped plaques of high signal intensity on T2WI/FLAIR involving mainly in the brain and spinal cord associated with left deep periventricular frontal and left cerebellar peduncle enhanced plaques as shown in Figures 3(a)–3(e). The cerebrospinal fluid revealed oligoclonal, while the rest of the parameters were within the normal limits.

He was treated with IV pulse dose steroids 1 g for 5 days with complete recovery. He was kept on NSAIDS for his ankylosing spondylitis and was recommended to start rituximab as a disease-modifying treatment to control multiple sclerosis. The patient left to his home country and was lost to follow-up.

## 3. Discussion

The development of CNS demyelination with anti-TNF-*α* therapy has been shown in several reports from various countries [5, 7, 8]. It is unclear, however, whether these demyelinating events are coincidental or whether they are causally associated with the use of TNF-*α* antagonists [7]. Clinical trials of TNF-*α* blockers in patients with MS showed an increase in demyelinating events [9], in addition to the complete remission of symptoms after withdrawal of this agent [5, 7] and temporal relation, as well as the rechallenge phenomenon that was demonstrated in many cases [10–13] favor a causal association.

On the other hand, one wonders whether this correlation is simply the unmasking of a preexisting MS or the onset of new demyelinating diseases [5, 7, 8]. Interestingly, some authors identified the demyelination of the CNS in asymptomatic patients before the initiation of anti-TNF-*α* therapy, which suggests that TNF-*α* blockers may unmask preexisting MS as well as provoking new-onset demyelination [5].

The mechanisms underlying demyelination predisposition or demyelination exacerbation in patients treated with anti-TNF-*α* agents are not fully understood, and several theories have been suggested. One of these theories is called lack of entry theory, which suggests that TNF-*α* blockers cannot penetrate the intact blood-brain barrier (BBB) to suppress demyelination, but they can enhance demyelination through increased ingress of peripheral autoreactive T cells into the CNS [8]. This theory provides a possible explanation for the failure of anti-TNF-*α* blockers in reducing demyelination and for their effect on aggravating MS. However, they cannot adequately explain the new onset of demyelination, as in our cases.

The time between exposure to TNF-*α* blockers and the onset of symptoms varies widely between different reports. In the report by Kemanetzoglou et al. [8], the time between exposure to TNF-*α* blockers and the onset of symptoms ranged between 3 days and 6 years, while Mohan et al. [11] found that the time between exposure and the onset of symptoms was between 1 week and 15 months. As noted in our series, the time between the initiation of TNF-*α* blocker and the development of symptoms ranged between 2 years as in cases 1 and 2 and 5 years as in case 3, which falls within the range of 3 days to 6 years that was mentioned above.

As noted in our series and other reports [5, 7, 8], the discontinuation of TNF-*α* blockers led to a complete remission of symptoms. Relapses of demyelination after remission have been reported by many authors [14, 15] as well as in case 3 of our series, which could be explained by the common spontaneous relapsing-remitting episodes of MS. However, despite discontinuation of treatment, demyelination disorders may persist, indicating that anti-TNF-*α* agents may activate the demyelination mechanism, which further develops independently [15]. Our small number of cases despite the increased use of TNF-*α* blockers is reassuring and similar to other recently published data from the British registry [16]. Another question to be answered is whether demyelination during anti-TNF-*α* agents actually meets the criteria for MS diagnosis or whether it constitutes a different MS-like syndrome. From our study, it is recommended to avoid the use of TNF-*α* blockers in patients with own history or family history of MS or any other demyelinating disease. In case of development of any new neurological symptoms, the patient needs a thorough neurological examination, and once demyelination is confirmed, anti-TNF blockers should be withdrawn. In addition to those with a family history of demyelination, those with a family history of autoimmune disease might also be at higher risk of developing demyelinating events as in our first case, even in the absence of TNF-*α* blockers due to shared common gentic and environmental triggers [17].

## 4. Conclusion

Despite the small number of patients, our series adds to the growing body of evidence supporting a causal link between anti-TNF-*α* agents and demyelination, which clinicians should be aware of. On expectation of any neurological side effects, early discontinuation of TNF-*α* blockers and requesting urgent MRI scan to confirm the diagnosis is of utmost importance.

## Data Availability

No data were used to support this study.

## Abbreviations

Anti-TNF-*α:*: Tumor necrosis factor antagonists
MRI: Magnetic resonance imaging
MS: Multiple sclerosis
CSF: Cerebrospinal fluid
BBB: Blood-brain barrier.
